# Pregnancy coercion and partner knowledge of contraceptive use among Ethiopian women

**DOI:** 10.1016/j.conx.2022.100084

**Published:** 2022-09-17

**Authors:** Jessica L. Dozier, Linnea A. Zimmerman, Bedilu A. Ejigu, Solomon Shiferaw, Assefa Seme, Mahari Yihdego, Robel Yirgu, Shannon N. Wood

**Affiliations:** a Department of Population Family and Reproductive Health, Johns Hopkins Bloomberg School of Public Health, Baltimore, MD, United States; b Department of Statistics, College of Natural Sciences, Addis Ababa University, Addis Ababa, Ethiopia; c School of Public Health, Addis Ababa University, Addis Ababa, Ethiopia; d Performance Monitoring for Action Ethiopia, Addis Ababa, Ethiopia

**Keywords:** Couples, Contraception, Covert use, Men, Pregnancy coercion, Reproductive coercion

## Abstract

**Objective:**

To examine the relationship between pregnancy coercion and partner knowledge of contraceptive use.

**Study design:**

Cross-sectional Performance Monitoring for Action-Ethiopia data were collected in October-November 2019 from a nationally representative sample of women ages 15 to 49. The analytical sample (*n* = 2,469) included partnered women using contraception in the past year. We used multinomial logistic regression to examine associations between past-year pregnancy coercion (none, less severe, more severe) and partner knowledge/couple discussion of contraceptive use (overt use with couple discussion before method initiation (reference group), overt use with discussion after method initiation, and covert use of contraception).

**Results:**

Most women reported their partner knew they were using contraception and had discussed use prior to method initiation (1,837/2,469, 75%); 16% used overtly and discussed use after method initiation, and 7% used contraception covertly. The proportion of covert users increased with pregnancy coercion severity (4%_none_, 14%_less severe_, 31%_more severe_), as did the proportion of overt users who delayed couple contraceptive discussions, (14%_none_, 23%_less severe_, 26% _more severe_); however, overt use with couple discussion before method initiation decreased with pregnancy coercion severity (79%_none_, 60%_less severe_, 40%_more severe_). The risk of covert use among women experiencing less severe pregnancy coercion was four times greater than women who experienced no pregnancy coercion (adjusted relative risk ratio, (aRRR) = 3.95, 95% confidence interval (CI) 2.20–7.09) and ten times greater for women who experienced the most severe pregnancy coercion (aRRR = 10.42, 95% CI 6.14–17.71). The risk of overt use with delayed couple discussion also increased two-fold among women who experienced pregnancy coercion compared to those who did not (less severe aRRR = 2.05, 95% CI 1.39–2.99; more severe aRRR = 2.89, 95% CI 1.76–4.73).

**Conclusion:**

When experiencing pregnancy coercion, women may avoid or delay contraceptive conversations with their partners. Increased pregnancy coercion severity has the greatest association with covert use and couple contraceptive discussions.

**Implications:**

The presence and timing of couple discussions about contraception are critical for ensuring safety for women experiencing pregnancy coercion. Screening for pregnancy coercion must be included within contraceptive counseling so that women can choose methods that maximize their reproductive autonomy.

## 1 Introduction

While violence against women occurs across socioecological levels, the partner dyad is a crucial influence on women's reproductive health given couple decision-making surrounding contraceptive use and childbearing [1,2]. Supportive male partners may improve reproductive outcomes, including uptake of contraception [3,4]; gender inequalities and unsupportive partners can impede women's ability to make reproductive decisions and lessen their autonomy [5,6]. Thus, to ultimately attain gender equality, concerted attention in addressing partner constraints on women's reproductive autonomy is essential [7].

One such constraint is partner-perpetrated reproductive coercion [8], [9], [10]. This form of intimate partner violence, where men interfere in reproductive decisions through direct contraceptive interference or pressure, includes the sub-forms of pregnancy coercion and condom manipulation [8,11]. Pregnancy coercion focuses on male behaviors encouraging pregnancy despite female fertility desires and intentions, whereas condom manipulation is specific to condom removal or sabotage [9,11]. Coercion from partners can adversely impact women's reproductive health outcomes; previous evidence suggests reproductive coercion is associated with decreased contraceptive use [12], [13], [14] and increased risk of unintended pregnancy [9,11,13].

One potential strategy that women use when facing reproductive coercion is contraceptive use without their partner's knowledge of use, called covert use. Quantitative studies on couple communication surrounding contraception generally focus on whether contraception was discussed or whether the partner knows of contraceptive use (i.e., the direct measure of covert use) [15], despite qualitative evidence indicating that disclosing contraceptive use after method initiation may also be risky [16]. Covert use is common in sub-Saharan Africa—a study using Demographic Health Survey data, including 14 countries from Sub-Saharan Africa, found direct estimates as high as 15% in Uganda and indirect estimates ranging from 10% in Uganda to 69% in Nigeria [15].

Despite the clear linkages between reproductive coercion and contraceptive use [9,10,12,14,17], there has been limited research on reproductive coercion and covert use of contraception in low- and middle-income countries. This association is pertinent to explore in Sub-Saharan Africa, where patriarchal gender norms remain prominent and where men's authority in decisions around fertility often overrides women's autonomy [7]. In Ethiopia, men hold significant authority over contraceptive decision-making [18,19], and women may hide contraceptive use due to male pressure to conceive, patriarchal gender norms, and power imbalances [5,16,17]. The prevalence of covert use of contraception among Ethiopian women ranges from 7% for direct estimates and 33% for indirect estimates [15]. Moreover, one in five Ethiopian women experiences pregnancy coercion —corresponding to a 30% reduction in the odds of contraceptive use [12].

The limited evidence examining the link between reproductive coercion and covert use of contraception suggests that partner coercion increases risk of covert use [17,20]. However, studies have generally been conducted among highly specific sub-groups, with no exploration among nationally representative samples. A study among survivors of intimate partner violence in Kenya highlights the existence of a potentially cyclic relationship between reproductive coercion and covert use, where women experiencing reproductive coercion attempted using contraception covertly multiple times before they were able to utilize it successfully [20]. Covert use may thus be a way for women to avoid discord or violent repercussions when faced with discordant fertility intentions and partner opposition to contraception [16,17,20]. However, partner discovery of covert use may also carry physical, financial, and social consequences, and women may discontinue use upon detection [16]. Few studies have examined nuanced definitions of covert and overt use of contraception, relying only on a binary distinction of partner knowledge that masks aspects of timing and communication. These measures may thus underestimate safety risks to women, particularly for those experiencing reproductive coercion [17,20].

The present study seeks to examine the association between reproductive coercion and partner knowledge of contraceptive use when considering the timing of couple communication about use in Ethiopia. Pregnancy coercion is the only form of reproductive coercion examined in the present study given requirement of partner knowledge in condom use and lack of data on condom manipulation from the parent study. Specifically, we examine the relationship between pregnancy coercion and partner knowledge about contraception (overt use with couple discussion before method initiation, overt use with no discussion, overt use with couple discussion after initiation, and with covert use of contraception) within a nationally representative sample of Ethiopian women.

## 2 Material and methods

### 2.1 Study design

Data come from Performance Monitoring for Action (PMA)-Ethiopia, which is a five-year research partnership (2019–2023) between Addis Ababa University, The Ethiopian Federal Ministry of Health, and Johns Hopkins Bloomberg School of Public Health [21]. We utilize data from nationally representative cross-sectional female and household surveys conducted among women aged 15 to 49 from October to November 2019. PMA-ET uses a two-stage cluster design, with data collected in each of nine regional and two administrative states. A total of 264 enumeration areas and 35 households in each enumeration areas were randomly selected. Women were eligible for the female survey if they were age 15 to 49, lived in the selected enumeration area boundaries, and slept in the selected household the night before. All participants provided oral informed consent. Surveys took approximately 30 to 60 min to complete.

Data were collected by female resident enumerators utilizing a two-stage sampling design in six regions that cover 91% of the country's population. Trained resident enumerators collected data using mobile phones. Training addressed asking sensitive questions and ensuring participant confidentiality. Study procedures followed best practices for research on violence against women [22] and included facilitated referral to local health centers. Institutional Review Boards at the Bloomberg School of Public Health and Addis Ababa University approved study procedures. Full study procedures are available elsewhere [23].

### 2.2 Analytic sample

Pregnancy coercion items were asked exclusively to women who were married or living with a partner (*n* = 5605). As these analyses focus on overt and covert contraceptive use, the analytic sample was restricted to current and recent (past year) users of modern contraception (*n* = 2505). Women were further excluded if they were missing pregnancy coercion (*n* = 26) or covert use data (*n* = 12), for a final analytic sample of 2469 women.

### 2.3 Measures

The outcome variable of interest, partner knowledge/discussion of contraceptive use, was measured by combining two items: (1) the direct covert use item: "Does your husband/partner know that you are/were using METHOD?" and (2) an additional item to assess timing: "Did you talk with your partner about using [CURRENT/MOST RECENT METHOD?] before you started using, after you started using, or have you not talked about it?" This combination created a four-response categorical outcome variable: (1) overt use with discussion before use, (2) overt use with no discussion, (3) overt use with discussion after use, and (4) covert use.

Experience of past-year pregnancy coercion was measured via the five-item sub-scale from the Reproductive Coercion Scale [9,24]. Items included are "In the past 12 months, has your husband/partner (1) told you not to use family planning, (2) said he would leave you if you didn't get pregnant, (3) told you he would have a baby with someone else if you didn't get pregnant, (4) took away your family planning or kept you from getting to the clinic, and (5) hurt you physically because you did not agree to get pregnant?" Psychometric analyses indicated one latent construct (eigenvalue = 1.84) with moderate reliability (Cronbach's alpha = 0.69) [12].

Past-year pregnancy coercion was measured as a categorical variable (none, less severe, and more severe). An affirmative response to item one only (told you not to use family planning) was coded as less severe pregnancy coercion; more severe pregnancy coercion included affirmative responses to any item two through five. The categorical pregnancy coercion variable intended to differentiate ambiguous behaviors from those hypothesized to be more harmful with specified coercive intent [12].

Covariates included in the analysis were residence, age, marital status, education, number of children, religion, polygyny, and partner's age. Covariates were examined in binary or categorical form, with small groups (*n* < 20) combined, when possible, to maximize statistical power. The average score from contraceptive existence of choice sub-scale of the Women's and Girl's Empowerment-Sexual and Reproductive Health Index was also included [5,25]. Responses were recorded on a 5-point Likert scale and analyzed as continuous [25].

### 2.4 Analysis

Exploratory analyses were conducted to understand distributions of key variables. Design-based F-statistics were employed to test differences between sociodemographic variables and partner knowledge of contraceptive use categories. Unadjusted and adjusted multinomial logistic regression analyses were used calculate relative risk ratios (RRR) between outcome categories, with the base outcome defined as overt use with discussion before use. Due to small cell sizes (55/2469, <3%), overt use without partner discussion was excluded from multinomial models. Adjusted analyses accounted for covariates with *p* < 0.10 in unadjusted models. Sensitivity analyses were also conducted restricting the sample to current contraceptive users only (*n* = 1945), excluding past-year users of contraception (*n* = 522); results for past-year users only were not possible due to small sub-samples. All analyses were conducted in Stata 16 [26], with statistical significance set a priori at *p* < 0.05 and accounting for sampling weights.

## 3 Results

**Fig. 1:**
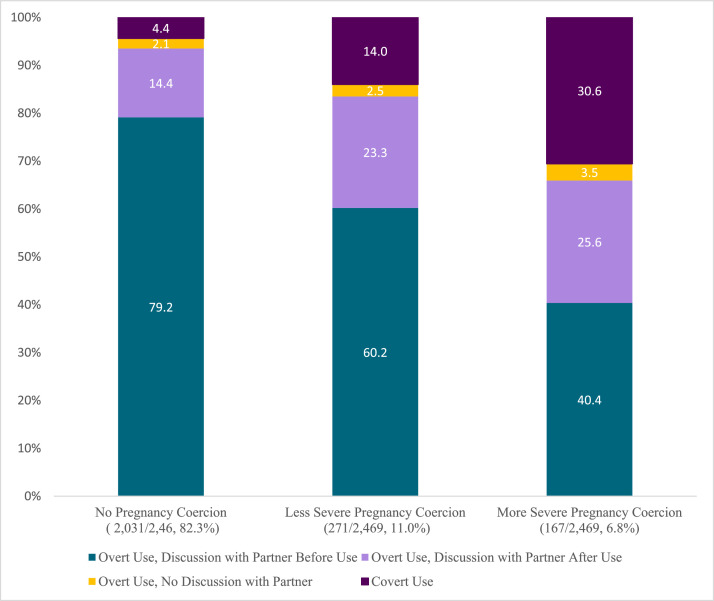
Partner knowledge of women's contraceptive use by experience of pregnancy coercion—Performance Monitoring for Action-Ethiopia, 2019 (*n* = 2469).

Characteristics of the analytic sample by outcome are shown in Table 1. Approximately 93% of currently partnered, recent contraceptive users reported using contraception overtly, and 7.2% reported covert use. About three-quarters of overt users reported discussing use with their partner before method initiation. Only 2.2% of overt users said their partner was aware of their contraceptive use, but they had not discussed it. Of overt users, 399/2291 (16%) reported discussing contraceptive use with their partners after method initiation. Overall, 17.7% of the sample reported experiencing any pregnancy coercion, 11.0% experienced less severe pregnancy coercion, and 6.8% experienced more severe pregnancy coercion. Women who experienced pregnancy coercion were more likely than those who did not to use contraception without their partner's knowledge or to use overtly with delayed couple discussion; the proportion of covert users and overt use with delayed couple discussion increased with pregnancy coercion severity (Fig. 1).

**Table 1:** Weighted sample characteristics for partnered women using contraception in the past 12 months by partner knowledge/couple discussion of contraception—Performance Monitoring for Action-Ethiopia, 2019 (*N* = 2469).

		Partner knowledge/couple discussion of contraception				
	Total	Covert use	Overt use, discussion with partner before use	Overt use, no discussion with partner	Overt use, discussion with partner after use	*p*-value^a^
*n* (row%)						
	2469 (100)	178 (7.2)	1837 (74.4)	55 (2.2)	399 (16.2)	
*Independent variable*						
Pregnancy coercion						**<0.001**
None	2031 (82.3)	89 (4.4)	1608 (79.2)	43 (2.1)	292 (14.4)	
Less severe	271 (11.0)	38 (14.0)	162 (59.9)	7 (2.5)	64 (23.7)	
More severe	167 (6.8)	51 (30.6)	67 (40.4)	6 (3.5)	43 (25.6)	
*Community characteristics, n* (row%)						
Residence						**<0.001**
Urban	851 (34.5)	23 (2.7)	664 (78.1)	18 (2.2)	146 (17.1)	
Rural	1618 (65.5)	155 (9.6)	1173 (72.5)	37 (2.3)	253 (15.7)	
*Woman's characteristics*						
Age (in y)						**0.008**
15–19	172 (7.0)	17 (9.7)	126 (73.4)	0 (0)	29 (16.9)	
20–29	1196 (48.4)	53 (4.4)	927 (77.5)	24 (2.0)	192 (16.0)	
30–39	821 (33.3)	71 (8.6)	591 (72.0)	22 (2.7)	138 (16.8)	
40–49	280 (11.3)	38 (13.4)	193 (69.0)	9 (3.2)	40 (14.4)	
Marital status						0.09
Married	2379 (96.3)	168 (7.1)	1782 (75.0)	51 (2.2)	376 (15.8)	
Living with a partner	90 (3.7)	10 (10.3)	54 (60.2)	4 (4.3)	23 (25.2)	
Education						**<0.001**
Never attended	910 (36.9)	93 (10.2)	639 (70.2)	31 (3.4)	147 (16.3)	
Primary	952 (38.6)	66 (7.0)	695 (73.0)	19 (2.0)	171 (18.0)	
Secondary or higher	607 (24.6)	18 (3.0)	503 (83.0)	5 (0.9)	79 (13.1)	
Number of children						**<0.001**
None	242 (9.8)	8 (3.2)	182 (75.3)	1 (0.2)	52 (21.4)	
1 child	572 (23.2)	35 (6.1)	430 (75.3)	5 (0.9)	102 (17.8)	
2–3 children	800 (32.4)	39 (4.9)	611 (76.4)	23 (2.8)	127 (15.9)	
4+ children	855 (34.7)	96 (11.3)	614 (71.9)	27 (3.2)	118 (13.8)	
Religion						**0.006**
Orthodox	1325 (53.7)	79 (6.0)	967 (73.0)	32 (2.5)	246 (18.6)	
Muslim	442 (17.9)	57 (12.8)	315 (71.3)	7 (1.7)	63 (14.3)	
Protestant	646 (26.2)	38 (5.9)	513 (79.5)	14 (2.1)	80 (12.4)	
Other^b^	56 (2.3)	3 (5.7)	41 (74.4)	2 (2.7)	10 (17.1)	
Contraceptive existence of choice score, mean (SD)^c^	3.95 (0.8)	3.53 (0.8)	4.02 (0.05)	3.5 (0.2)	3.9 (0.06)	**<0.001**
*Partner's characteristics*						
Husband/Partner's number of wives						**0.001**
Monogamous	2292 (92.9)	152 (6.6)	1730 (75.5)	47 (2.1)	363(15.8)	
Polygynous	176 (7.2)	26 (14.7)	104 (60.8)	8 (4.4)	36 (20.2)	
Husband/Partner's age (in y)						**<0.001**
15–29	676 (27.4)	24 (3.6)	527 (78.1)	11 (1.6)	113 (16.8)	
30–39	932 (37.7)	57 (6.1)	716 (76.9)	20 (2.2)	138 (14.9)	
40–49	553 (22.4)	46 (8.4)	405 (73.5)	17 (3.1)	84 (15.0)	
50+	308 (12.5)	50 (16.2)	187 (61.0)	7 (2.3)	63 (20.5)	

a Design-based F-statistic; Bold indicates p<0.05.

b Includes Catholic, Traditional, Wakefeta, Non-believers, and Other.

c Responses range from strongly agree to strongly disagree on a 5-point Likert scale, with higher scale scores indicating greater existence of choice (i.e., motivational autonomy).

All covariates were significantly associated with the outcome categories (Table 1). The proportion of covert users increased with higher parity and partner age. Conversely, the proportion of covert users decreased with increasing women's education. Women who reported overt use with couple discussion before use had the greatest contraceptive existence of choice scores. Rural residence was associated with greater covert use compared to urban residence. Similarly, women in the youngest and oldest age categories reported greater covert use than other age groups.

Multinomial regression results are shown in Table 2; the aRRR presented compare partner's knowledge of contraceptive use categories to overt users who discussed contraceptive use with their partners prior to method initiation (reference group). The risk of overt use with discussion after method initiation doubled among women who experienced less severe pregnancy coercion (aRRR = 2.05, 95% confidence interval (CI) 1.39–2.99) and nearly tripled among those who experienced more severe pregnancy coercion (aRRR = 2.90, 95% CI 1.76–4.73) compared to women who did not experience pregnancy coercion. The risk of covert use among women experiencing less severe pregnancy coercion was four times greater than women who experienced no pregnancy coercion (aRRR = 3.95, 95% CI 2.20–7.09) and ten times greater for women who experienced the most severe pregnancy coercion (aRRR = 10.42, 95% CI 6.14–17.71).

**Table 2:** Adjusted Multinomial logistic regression of pregnancy coercion on partner knowledge/couple discussion of Ethiopian women's contraceptive use (overt use with discussion before use (base outcome), overt use with discussion after method initiation, overt use with no couple discussion about use, and covert use^a^)—Performance Monitoring for Action-Ethiopia, 2019 (*N* = 2469).

	Partner knowledge/couple discussion						
	Overt use, discussion with partner *before* use (base outcome)	Overt use, discussion with partner *after* use versus overt use, discussion with partner *before* use		Overt use, no discussion with partner versus overt use, discussion with partner *before* use		Covert use versus overt use, discussion with partner *before* use	
		aRRR^b^	95% CI	aRRR^b^	95% CI	aRRR^b^	95% CI
Pregnancy coercion							
None (reference)		–	–	–	–	–	–
Less severe	–	**2.05**	**(1.39–2.99)**	1.55	(0.45–5.30)	**3.95**	**(2.20–7.09)**
More severe	–	**2.90**	**(1.76–4.73)**	2.19	(0.62–7.75)	**10.42**	**(6.14–17.71)**
Residence							
Rural (reference)	–	–	–			–	
Urban	–	1.04	(0.73–1.48)	1.78	(0.78–4.04)	**0.38**	**(0.23–0.63)**
Age (in years)							
15–19 (reference)	–	–	–	–	–	–	–
20–29	–	1.23	(0.64–2.39)	a	a	**0.28**	**(0.09–0.88)**
30–39	–	1.76	(0.78–4.00)	a	a	0.37	(0.10–1.36)
40–49	–	1.20	(0.47–3.07)	a	a	0.35	(0.09–1.37)
Marital status							
Married (reference)	–	–	–	–	–	–	
Living with partner	–	**1.79**	**(1.02–3.14)**	2.79	(0.57–13.77)	2.83	(0.70–11.46)
Education							
Never attended (reference)	–	–	–	–	–	**–**	–
Primary	–	0.98	(0.68–1.42)	0.66	(0.32–1.39)	0.87	(0.55–1.39)
Secondary or higher	–	**0.61**	**(0.39–0.96)**	**0.30**	**(0.10–0.90)**	0.84	(0.42–1.72)
Number of children							
Nulliparous (reference)		–	–	–	–	–	–
1 child	–	0.97	(0.54–1.74)	5.79	(0.81–41.3)	**4.23**	**(1.29–13.88)**
2–3 children	–	0.74	(0.40–1.38)	**17.5**	**(2.79–109.4)**	3.30	(0.96–11.33)
4+ children	–	0.49	(0.23–1.04)	**20.1**	**(2.55–159.0)**	3.81	(0.96–15.11)
Religion							
Orthodox (reference)	–	–	–	–	–	–	–
Muslim	–	0.80	(0.49–1.30)	0.67	(0.23–1.98)	**2.08**	**(1.25–3.43)**
Protestant	–	**0.64**	**(0.44–0.92)**	0.77	(0.35–1.69)	0.80	(0.46–1.39)
Other	–	1.09	(0.54–2.20)	1.18	(0.14–9.67)	1.01	(0.21–4.73)
Husband/partner's number of wives							
Monogamous (reference)	–	–	–	–	–	–	–
Polygamous	–	1.59	(0.98–2.60)	2.01	(0.69–5.89)	1.93	(0.96–3.89)
Husband/partner's age (in y)							
15–29 (reference)	–	–	–	–	–	–	–
30–39	–	0.93	(0.64–1.34)	0.69	(0.26–1.89)	1.78	(0.84–3.74)
40–49	–	1.01	(0.59–1.73)	0.93	(0.29–2.98)	1.78	(0.75–4.23)
50+	–	**1.82**	**(1.05–3.17)**	0.90	(0.26–3.17)	**4.54**	**(2.23–9.24)**
Contraceptive existence of choice score	–	0.85	(0.72–1.00)	**0.48**	**(0.30–0.75)**	**0.66**	**(0.50–0.87)**

Notes. The adjusted relative risk ratios (aRRR) presented compare husband/partner's knowledge of contraceptive use categories to overt users who discussed contraceptive use with their partners prior to method initiation (base outcome). Bold indicates p<0.05.

a Overt Use, no discussion with partner categories not shown due small cell sizes (55/2469).

b Adjusted model includes pregnancy coercion, residence, women's age, marital status, women's education, number of children, religion, polygyny, partner's age, and mean contraceptive existence of choice sub-scale score from the Women's and Girl's Empowerment-Sexual and Reproductive Health (WGE-SRH) index.

Similar directionality and associations were observed when the sample was restricted to current contraceptive users only in sensitivity analyses (not shown). The risk of covert use among women who experienced more severe pregnancy coercion in the past year was almost 18-fold greater (aRRR = 17.91, 95% CI: 9.72–32.99) relative to those who did not experience pregnancy coercion. The risk of covert use among women who experience less severe pregnancy coercion was six times greater than those who experienced none (aRRR = 6.15, 95% CI: 2.95–12.81).

## 4 Discussion

This study examines the association between pregnancy coercion and partner knowledge of contraceptive use when considering timing of couple communication about contraception. We found past-year pregnancy coercion was associated with increased risk of overt contraceptive use with couple conversations occurring after method initiation. These findings highlight that women may avoid or delay contraceptive use discussions with their partners when experiencing pregnancy coercion. While both less severe and more severe pregnancy coercion were associated with timing and presence of couple contraceptive discussions, more severe pregnancy coercion was associated the greatest risk. This suggests avoiding or delaying contraceptive discussions with partners may be an important women-implemented safety strategy when faced with partner control of known coercive intent. Thus, understanding the presence of couple discussions and the timing of these discussions is critical for promoting women's reproductive autonomy.

Partner communication about contraception may suggest a more equitable relationship between a woman and her partner; however, as these results suggest, discussions may not be feasible for all women. If a partner is known to oppose contraception, women may choose to delay or avoid couple discussion, as seen from results revealing heightened risk of overt use with delayed discussion and covert use for women who have experienced reproductive coercion in the past year. Our findings on covert use and pregnancy coercion are consistent with previous studies from Niger among married adolescents [17] and in Kenya among women experiencing intimate partner violence [20], which suggest an increased risk of covert use among women experiencing reproductive coercion. Further, while both less severe and more severe forms of pregnancy coercion were associated with delayed and omitted partner discussions, most severe forms displayed the highest risks, indicating that known coercive intent and violence could be potent inhibitors in holding contraceptive discussions. Both delay and omission may serve as important strategies for women to protect themselves from violence and concurrently meet their reproductive preferences.

Of note, some women reported using contraception overtly, without any partner discussion. Limited evidence indicates that male partners may be aware of their partner's contraceptive use without ever having explicit couple conversations due to beliefs that contraception is not a man's responsibility [16]. Further research with larger sample sizes is needed to understand the contexts of these conversations and when it is valuable, irrelevant, or harmful to engage men in contraceptive use decisions.

This study is not without limitations. Foremost, the cross-sectional nature of these data limits our understanding of the temporality between pregnancy coercion and partner knowledge of contraception. Women may either be using contraception covertly given pregnancy coercion experience or be at greater risk for pregnancy coercion due to covert use, as suggested by qualitative data from Kenya indicating a potentially cyclic relationship [20]. Further, this study utilizes women's reports of male behaviors and includes limited male covariate data. Inclusion of measures from both partners would facilitate a more nuanced understanding of dynamics, conversations, and motivations. Future research should seek to include more partner sociodemographic and attitude data.

Approximately 20% of women experience pregnancy coercion in Ethiopia [12], and providers must be aware of its impact on contraceptive use and continuation [27]. Women may actively conceal their use of contraception from partners when experiencing violence and coercion, allowing them to circumvent male control. Thus, covert use could be a valuable safety strategy for women to protect themselves against pregnancy coercion and its consequences, including unintended pregnancy [20]. Contraceptive counseling should include screening for pregnancy coercion and discussions on the nature of partner involvement in reproductive decision-making [11,28]. Counseling strategies should also ensure that women can select methods that can be used discreetly, if they choose. Providers should work with women to identify the most appropriate and safest approaches to engaging men in contraceptive and reproductive decisions.

Taken together, our results indicate that for women experiencing pregnancy coercion, both the presence and timing of couple discussions about contraception are critical. Inclusion of the direct covert use measure [15], along with the expanded timing of couple discussion measure, in population-based surveys like the Demographic and Health Surveys and PMA, is needed to capture partner involvement more thoroughly. Longitudinal research, including work with men and boys, is needed to disentangle complex pathways surrounding partner dynamics and assess the impact of pregnancy coercion on method continuation. The integration of pregnancy coercion screening and discussion of the role partners play in decision-making, including as barriers or facilitators to use, is necessary to ensure women's safety. Equipping family planning providers with the tools to help women make the best decisions for their specific situations and cultivating reproductive autonomy should be the ultimate priority.

## Data availability statement

Data are available upon request at pmadata.org.
